# Health‐related quality of life and fear of progression in individuals with Li‐Fraumeni syndrome

**DOI:** 10.1002/jgc4.1859

**Published:** 2024-02-13

**Authors:** Senta Kiermeier, Sarah Schott, Juliane Nees, Christina Dutzmann, Farina Strüwe, Christian P. Kratz, Christina Sauer, Anna Fleischer, Myriam Keymling, Imad Maatouk

**Affiliations:** ^1^ Section of Psychosomatic Medicine, Psychotherapy and Psychooncology, Department of Internal Medicine II Julius‐Maximilian University Würzburg Würzburg Germany; ^2^ Department of Gynecology and Obstetrics University Hospital Heidelberg Heidelberg Germany; ^3^ Department of Pediatric Hematology and Oncology Hannover Medical School Hannover Germany; ^4^ Department of General Internal Medicine and Psychosomatics University Hospital Heidelberg Heidelberg Germany; ^5^ Department of Radiology German Cancer Research Center (DKFZ) Heidelberg Germany

**Keywords:** distress, fear of progression, Li‐Fraumeni syndrome, mental health, psychosocial, quality of life

## Abstract

Li‐Fraumeni syndrome (LFS) is a rare autosomal dominant cancer predisposition syndrome associated with a highly elevated lifetime cancer risk. This and the recommended intense surveillance program represent a large psychological burden on families. In order to develop targeted psychosocial interventions, we conducted a needs assessment. Adults (≥18 years) with LFS were included via regular hospital visits and online support groups and newsletters. Individuals filled out a questionnaire addressing among others: fear of progression (FoP‐questionnaire, short‐form), health‐related quality of life (HRQoL, Short‐Form Health Survey‐12), distress (National Comprehensive Cancer Network distress thermometer), perceived cancer risk, and aspects of surveillance adherence. Collecting data over a 14‐month period (March 2020 – June 2021), 70 adults were recruited (female = 58, 82.9%; mean age = 41.53 years). With mean mental component scores (MCS) of 42.28 (*SD* = 10.79), and physical component scores (PCS) of 48.83 (*SD* = 10.46), HRQoL was low in 34.8% (physical) and 59.4% (mental) of individuals when applying a mean cut‐off of 45.4 (PCS) and 47.5 (MCS) to indicate poor HRQoL. High levels of FoP and distress were present in 68.6% and 69.1% of the participants, respectively. Performing a multiple linear regression on MCS and PCS, no sociodemographic variable was shown to be significant. FoP (*β* = −0.33, *p* < 0.05) and distress (*β* = −0.34, *p* < 0.05) were significantly associated with MCS. Individuals in our sample were burdened more than expected, with the majority reporting low levels of (mental) HRQoL, high distress, and FoP. Psychosocial support is necessary to help individuals with LFS (survivors as well as “previvors”) increase their HRQoL, as it is crucial to survival.

**What is known about this topic**

Li‐Fraumeni syndrome (LFS) is a condition associated with a high probability of developing cancer. As an autosomal dominant cancer predisposition syndrome, it affects both the physical and mental health of families intensively.

**What this paper adds to the topic**

We demonstrate that fear of cancer progression and distress were greater than expected in most participants, with impairment in terms of mental quality of life. We suggest that the psychosocial burdens of individuals with LFS need to be assessed regularly, as people with LFS clearly require focused support to deal with their fears, especially also in times of no acute cancer diagnosis.

## 1 INTRODUCTION

Li‐Fraumeni syndrome (LFS) is a rare autosomal dominant cancer predisposition syndrome (CPS) caused by (likely) pathogenic germline variants in the *TP53* gene (P/LP *TP53*) (Li et al., 1988). It was most recently defined as a spectrum, comprising four subtypes (phenotypic LFS, LFS, attenuated LFS, and incidental LFS) (Kratz et al., 2021). P/LP *TP53* variant carriers (VC) have a highly elevated lifetime risk of developing cancer. Risk estimation is challenging, as cancer penetrance varies according to mutation type, age, and sex (Amadou et al., 2018). Thus, different overall risk estimations are found in the literature, varying from 80% to nearly 100% of participants developing cancer by 70 years of age (Amadou et al., 2018; Mai et al., 2016). Owing to the fact that LFS is a dominantly inherited CPS, a striking familial cancer history is common, with an estimated range of only 7–20 percent for de novo cases (Jhaveri et al., 2015).

### 1.1 Psychological impact of LFS diagnosis and surveillance

Extensive surveillance, with its proven overall survival benefit, is recommended from birth onwards. While regular clinical or diagnostic check‐ups (Villani et al., 2011) are medically beneficial, they may also lead to anxiety, fear, skepticism, and distress (Rippinger et al., 2020; Ross et al., 2017). Individuals with LFS or other rare CPS with a high risk of developing multiple cancers exhibit moderate to clinically relevant levels of distress and a low health‐related quality of life (HRQoL) when under regular surveillance (Gopie et al., 2012; Rippinger et al., 2020). This indicates a possible trade‐off between medical and psychological well‐being in P/LP *TP53* VC (Jhaveri et al., 2015). As worrying is viewed as a possible barrier to adherence to cancer prevention programs, it is important to investigate the psychological well‐being of those living with LFS (Lammens, Bleiker, et al., 2010). P/LP *TP53* VC need to handle multiple psychosocial stressors in addition to surveillance. They often witness cancer and the death of loved ones along with their own cancer history (Oppenheim et al., 2001; Peterson et al., 2008). A further source of distress is when and how to undergo and communicate genetic testing, as well as deal with the familial cancer risk to their children (Alderfer et al., 2017; Valdez et al., 2017).

### 1.2 Fear of progression

Fear of progression (FoP, sometimes also termed “fear of recurrence”) is one of the most frequently identified issues in cancer care (Simard et al., 2013). It is defined as the fear of cancer developing, recurring, or progressing (Herschbach & Dinkel, 2014). It can occur in currently treated patients as well as survivors, regardless of cancer entities (van de Wal et al., 2016). Psychosocial interventions for FoP are necessary, as it can become a chronic condition, especially with high levels of FoP being associated with symptoms of depression, anxiety, or post‐traumatic stress (Pradhan et al., 2021). Knowledge of the likelihood of cancer recurrence has been found to influence FoP in cancer survivors (Pradhan et al., 2021). We therefore postulate that FoP is a pressing issue in individuals with LFS, considering their elevated lifetime cancer prevalence (Amadou et al., 2018). Especially noteworthy is the idea that P/LP *TP53* VC may suffer from FoP without necessarily ever being diagnosed with cancer.

To the best of our knowledge, this is the first study investigating FoP in adult P/LP *TP53* VC. In order to develop targeted psychosocial interventions, we need to improve our insight into how mental and physical HRQoL are impaired in this group and to what degree FoP, distress, and perceived cancer risk are relevant factors.

## 2 METHODS

### 2.1 Study design and participants

This cross‐sectional, observational study is part of a mixed‐method study to explore the psychosocial burden of people living with LFS. We collected data over a 14‐month period (March 2020 – June 2021). This article presents the quantitative psychosocial data of P/LP *TP53* VC. Data on participants' health behavior may be found elsewhere (Nees et al., 2022).

This study is an independent subproject of the German ADDRess alliance (translational research for persons with Abnormal DNA Damage Response, supported by the German Federal Ministry of Education and Research, BMBF) and we work in cooperation with the German cancer predisposition syndrome (CPS) registry, another subproject within the ADDRess alliance led by Hanover Medical School. Information on the research alliance and its subprojects may be found on the CPS registry website: https://www.krebs‐praedisposition.de/en. We approached patients during routine appointments at University Hospital Heidelberg and Hanover Medical School, and via previous studies if renewed contact had been agreed. The CPS Registry and the German‐speaking chapter of the LFS Association (www.lfsa‐deutschland.de) supported recruitment via their social media accounts and patient group on Facebook. Adult (≥18 years) P/LP *TP53* VC, regardless of the time point of their LFS diagnosis, cancer history, or current cancer situation, could participate after providing written informed consent. People with genetic variants of unclear significance or with mosaicism were not eligible to participate.

The study was approved by institutional review boards in Heidelberg (S‐017/2020) and Hanover (7233) and was conducted in accordance with the Declaration of Helsinki (2013). Informed consent was obtained from all patients prior to their inclusion in the study.

### 2.2 Study measurements

The research team developed a large survey on various psychosocial aspects of LFS, including validated questionnaires, open‐ended questions, and investigator‐designed questions. For this study, we focused on presenting only the results of the survey items listed in the following sections (80 items). The one‐time survey was made available as a paper or online version (provided by www.soscisurvey.de), dependent on participants' preference.

### 2.3 Sociodemographic variables

Sociodemographic variables included six investigator‐designed items on the topics of education level, children, employment, and marital status.

### 2.4 Variables related to LFS

We designed 14 items on LFS, personal and familial cancer history, and surveillance, including among others questions such as: “Have you ever been diagnosed with cancer?”, “Has one of your children ever been diagnosed with cancer?”, “How many siblings with a known LFS diagnosis do you have?”. We included information about the surveillance program provided by the German LFS Association and asked to what extent the respondent was following this recommended surveillance program and reasons for their (non‐)adherence. Participants were also asked to estimate their own risk of developing cancer or suffering a recurrence on a scale from 0% to 100%.

### 2.5 Fear of progression –short‐form

To assess FoP, we used the German 12‐item short‐form version of the fear of progression questionnaire (FoP‐Q‐SF) (Mehnert, Herschbach, et al., 2006). The FoP‐Q‐SF may be applied to populations with cancer, cancer survivors, chronic diseases, and partners matching the diverse cancer history of our target group (Peikert et al., 2021; Sarkar et al., 2014; Zimmermann et al., 2011). We adapted the questions by replacing “illness” with “LFS.” Items were scored on a 5‐point Likert scale (on which 1 = “never” and 5 = “very often”); the sum score could thus range from 12 to 60. In order to section the sample for low and high scores of FoP, regardless of cancer history, we used a cut‐off previously suggested for cancer patients, with a mean of ≥34 indicating a high level of FoP (Herschbach, Berg, et al., 2010).

### 2.6 Distress thermometer and problem list

The National Comprehensive Cancer Network Distress Thermometer (DT) and Problem List (PL) is a screening tool to identify sources of distress. We used the German version, validated for cancer patients (Mehnert, Müller, et al., 2006). Participants circled the number on a scale from 0 to 10 that described best how much distress they have been experiencing in the past week including the current day (0 = no distress, 10 = extreme distress). The PL comprises 35 binary (yes/no) items, placed into physical (20), spiritual (2), practical (5), emotional (5), family (2), and other (1) groups. The DT cut‐off score representing clinically elevated levels of distress is ≥5 (Mehnert, Müller, et al., 2006).

### 2.7 Short‐form health survey‐12

We used the German version of the short‐form health survey (SF‐12) to asses HRQoL (Drixler et al., 2020). The SF‐12 comprises 12 items, resulting in two sum scores: the physical component score (PCS) and mental component score (MCS). Raw scores were converted into T‐scores (mean = 50, *SD* = 10), ranging from 0 (lowest state of HRQoL) to 100 (highest state of HRQoL). A poor PCS was indicated by a score ≤ 45.4, poor MCS by a score ≤ 47.5. To our knowledge, there are no suggested cut‐offs for LFS or other CPS. We therefore relied on cut‐offs previously applied in a cohort of young adult cancer survivors (Harju et al., 2018). We described an analysis of HRQoL and health behavior in this cohort elsewhere (Nees et al., 2022).

### 2.8 Statistical analysis

For continuous variables, we calculated the mean value, standard deviation, minimum, and maximum. We used absolute and relative frequencies to describe categorical variables and determined Pearson's correlation. Cronbach's alpha was used as a measure of the internal consistency of survey items. With respect to missing data, we excluded one case with missing data in the SF‐12. If one item was missing in the FoP‐Q‐SF, the mean of the remaining items was used as the replacement (*n* = 7). Two multiple linear regression analyses on MCS and PCS were performed with demographic and LFS‐related variables (sex, age, education, marital status, cancer history, surveillance participation, and perceived cancer risk), distress, and FoP as criterion variables. To calculate the effect sizes of significant factors, we used Cohen's *f* ^2^, with a *f* ^2^ ≥ 0.35 indicating a high effect size (Selya et al., 2012). The significance level was set at *p* < 0.05 for all statistical tests, with 95% confidence intervals. The coefficient of determination *R* ^2^ was used to indicate the proportion of data explained by the multiple regression model and *β* as standardized regression coefficient. Prior to calculations, we performed list‐wise case exclusions. There was no evidence of any assumptions being violated.

## 3 RESULTS

### 3.1 Study cohort

Seventy P/LP *TP53* VC took part, with a mean age of 41.53 years (range: 22–69 years). The average participant was female (*n* = 58, 82.9%) and married (*n* = 41, 58.6%) (Table 1). The duration between survey participation and genetic testing was available for 44 participants and averaged 3.43 years since the resulting LFS diagnosis (*SD* = 3.32, range = 0–16).

**TABLE 1:** Characteristics of study cohort.

	*n*	Mean (%)
Age (mean/*SD*)	70	41.53 ± 12.11
Female	70	58 (82.9)
Highest school‐leaving qualification	69	
Basic school‐leaving certificate		8 (11.6)
Tenth grade		16 (23.2)
Vocational school entrance		7 (10.1)
Twelfth/thirteenth grade		9 (13.0)
Bachelor's, Master's, Doctorate		24 (34.8)
Other		5 (7.2)
Occupational status	70	
Student/apprentice		6 (8.6)
Stay‐at‐home parent		3 (4.3)
Employee or freelancer		50 (71.4)
Retired		6 (8.6)
Other		5 (7.1)
Insurance status	70	
Compulsory		65 (92.9)
Private		5 (7.1)
Marital status	70	
Single		14 (20.0)
In a relationship		11 (15.7)
Married		41 (58.6)
Divorced/separated		3 (4.3)
Widowed		1 (1.4)
Children		
Having children	70	39 (55.7)
Child(ren) with cancer history	39	9 (23.1)
Li‐Fraumeni syndrome/cancer history		
Personal cancer history	70	47 (67.1)
One or more sibling with diagnosis of Li‐Fraumeni syndrome	58	27 (46.5)
Surveillance participation	67	
Complete adherence to surveillance		37 (55.2)
No or partial adherence to surveillance		30 (44.8)

### 3.2 SF‐12, perceived cancer risk and fear of progression

Table 2 depicts the descriptive results of SF‐12, perceived cancer risk, and FoP‐Q‐SF. The majority of our participants presented high MCS, as well as high levels of FoP and distress (59.4%–69.1%). Furthermore, a majority of participants (69.1%) estimated that they were more likely to suffer from cancer than not. Cronbach's *alpha* for the FoP‐Q‐SF in this sample was *α* = 0.87 (0.88 for the standardized items). Table 3 presents means and standard deviations for each individual item of the FoP‐Q‐SF.

**TABLE 2:** Results on quality of life, fear of progression, distress and perceived cancer risk.

	*n*	Min	Max	Mean	*SD*	Possible range	Threshold	Threshold *n* (%)
Physical component score	69	17.59	64.45	48.83	10.46	0–100	≤45	24 (34.8%)
Mental component score	69	24.64	60.70	42.28	10.79	0–100	≤47	41 (59.4%)
FoP^a^ sum score	70	16.00	60.00	38.00	9.81	12–60	≥34	48 (68.6%)
Distress last week, including today	68	0	10	5.72	2.53	0–10	≥5	47 (69.1%)
Perceived cancer risk	67	15	100	71.78	22.74	0–100	≥50	47 (69.1%)

^a^ FoP = Fear of progression.

**TABLE 3:** Descriptive results of each item of the fear of progression questionnaire (short form).^a^

	Mean	*SD*
1. I become anxious if I think my LFS may progress.	3.36	1.10
2. I am nervous prior to doctors' appointments or periodic examinations.	3.43	1.33
3. I am afraid of pain.	2.89	1.29
4. I have concerns about reaching my professional goals because of my illness.	3.06	1.32
5. When I am anxious, I have physical symptoms, such as a rapid heartbeat, stomach ache, or agitation.	3.37	1.23
6. The question of whether my children might also get LFS worries me.	3.49	1.66
7. It disturbs me that I may have to rely on strangers for activities of daily living.	3.01	1.29
8. I am worried that at some point in time I will no longer be able to pursue my hobbies because of LFS.	2.63	1.27
9. I am afraid of severe medical treatments during the course of LFS.	3.40	1.18
10. I worry that my treatment could damage my body.	3.00	1.20
11. I worry about what will become of my family if something should happen to me.	3.61	1.24
12. The thought that I might not be able to work due to LFS disturbs me.	2.76	1.16

^a^ These items are a translation of the validated German version we used in the study.

### 3.3 Distress

The mean distress in our sample (*n* = 68, two missing values) was 5.72 (*SD* = 2.53, range: 0–10), with 69.1% (*n* = 47) reporting a value above 5. Table 4 summarizes how frequently a problem was reported; we only display the problems with which more than 20% of participants agreed for reasons of clarity. Out of 20 physical problems, 11 were reported by more than 20%. Overall, the most frequently reported problems were worries, fears, and fatigue, followed by nervousness, sleep, and sadness. Pearson's correlation revealed the sum of the physical and emotional problems as significant: *r* = 0.495, *p* ≤ 0.001.

**TABLE 4:** Results of the most mentioned problems of the NCCN distress thermometer problem list.

	*n* (*n* = 69)	%
Practical problems		
Insurance	14^a^	20.6
Work/school	23	33.3
Family problems		
Dealing with partner	20	29.0
Emotional problems		
Worry	51	73.9
Fears	47	68.1
Sadness	37^a^	54.4
Depression	17	24.6
Nervousness	39^a^	57.4
Physical problems		
Pain	33^a^	48.5
Nausea	16^a^	23.5
Fatigue	42^a^	61.8
Sleep	38	55.1
Getting around	19^a^	27.9
Appearance	16^a^	23.5
Indigestion	23^a^	33.8
Skin dry/itchy	24^a^	35.3
Tingling in hands/feet	19^b^	28.4
Feeling swollen	21^a^	30.9
Sexual	21^a^	30.9

^a^ *n* = 68.

^b^ *n* = 67.

### 3.4 Multiple linear regression

To conduct multiple linear regressions, we transformed our variables to include them as factors (Table 5). The data of 61 individuals were included. None of the sociodemographic variables was significantly associated with MCS or PCS in our sample. However, FoP (*β* = −0.33, *p* < 0.05) and distress (*β* = −0.34, *p* < 0.05) contributed significantly to MCS. The only factor to have any statistically significant association with PCS was cancer history, albeit only marginally (*β* = −0.28, *p* = 0.052). To calculate the effect sizes of significant factors, we used Cohen's *f* ^2^ (Selya et al., 2012). After performing linear regression on MCS, FoP and distress were shown to have the same low to medium effect (*f* ^2^ = 0.14). Cancer history had only a small effect on PCS (*f* ^2^ = 0.08). Table 5 lists a summary of the results. We also conducted a subgroup analysis, additionally including the time in years since LFS diagnosis (*n* = 41). Here, the model on MCS revealed a coefficient of determination *R* ^2^ (adjusted) of 0.36 (0.15) and, for PCS, an *R* ^2^ (adjusted) of 0.23 (−0.03). In each model, the time in years since LFS diagnosis and HRQoL was revealed to be negatively associated, albeit statistically insignificantly (MCS: *B* = −0.61, *SE(B)* = 0.55, *β* = −0.19, *p* = 0.28; PCS: *B* = −0.48, *SE(B)* = 0.55, *β* = −0.16, *p* = 0.39).

**TABLE 5:** Results of multiple linear regression analyses of predictors contributing to MCS and PCS.

Predictors^a^	HRQoL (MCS)				HRQoL (PCS)			
	*B* [95%‐CI]	*s.e. B*	*β*	*p*	*B* [95%‐CI]	*s.e. B*	*β*	*p*
Gender	1.67 [−5.79 to 9.14]	3.72	0.06	0.66	−1.21 [−9.30 to 6.89]	4.03	−0.04	0.77
Age	0.01 [−0.24 to 0.25]	0.12	0.01	0.95	−0.16 [−0.43 to 0.10]	0.13	−0.19	0.23
Education	−2.57 [−11.34 to 6.20]	4.37	−0.08	0.56	−0.25 [−9.76 to 9.26]	4.74	−0.01	0.96
Marital status	0.13 [−6.30 to 6.55]	3.12	0.01	0.97	−4.94 [−11.90 to 2.02]	3.47	−0.22	0.16
Cancer history	−2.01 [−7.70 to 3.69]	2.84	−0.09	0.48	−6.11 [−12.29 to 0.07]	3.08	−0.28	0.05
Surveillance participation	−2.14 [−7.30 to 3.02]	2.57	−0.10	0.41	0.866 [−4.73 to 6.46]	2.79	0.04	0.76
Perceived cancer risk	1.91 [−3.51 to 7.32]	2.70	0.08	0.48	−0.79 [−6.66 to 5.08]	2.92	−0.4	0.79
FoP	−0.367 [−0.65 to −0.08]	0.14	−0.33	0.01*	−0.03 [−0.33 to 0.28]	0.15	−0.03	0.87
Distress	−1.56 [−2.73 to −0.39]	0.58	−0.34	0.01*	0.14 [−1.13 to 1.41]	0.63	0.03	0.82
Explained Variance *R* ^2^		0.37				0.12		
Adjusted *R* ^2^		0.25				−0.04		

Abbreviations: FoP, fear of progression; HRQoL, Health‐related quality of life; MCS, mental component score; PCS, physical component score.

^a^ *Predictors*: marital status (married or committed relationship vs. other), education (10 years or more vs. fewer than 10 years), cancer history (have had cancer vs. none), surveillance participation (completely following the recommended surveillance program for Li‐Fraumeni syndrome vs. not or only partial), perceived cancer risk (perceived risk more than 50% vs. less than 50%).

* *p* < 0.05.

## 4 DISCUSSION

This study is the first to investigate FoP and distress as well as their contribution to health‐related quality of life (HRQoL) in *TP53* variant carriers (VC). The majority of participants were notably burdened as they exhibited low mental component scores (MCS) (59.4%), a strong fear of progression (FoP) (68.6%), and a high level of distress (69.1%). Approximately one‐third of participants (34.8%) exhibited poor physical health and around two‐thirds (67.1%) revealed having had cancer at least once. This was only barely significantly associated with the physical component score (PCS). Most participants (69.1%) assumed the probability of developing cancer or suffering a recurrence was higher than 50%. Despite an overall poor model fit, FoP and distress were significantly associated with MCS. Our results imply that there is an association between the time since LFS diagnosis in an individual and increased impairment of their HRQoL.

### 4.1 Fear of progression

Our cohort scored essentially higher in each single item as well as overall in the FoP‐questionnaire, compared to other cancer survivors without LFS or any other form of CPS (Hanprasertpong et al., 2017; Koch et al., 2013; Mehnert et al., 2009). The knowledge of increased personal cancer risk, the lack of causal therapy, and the resulting need for regular screening examinations may lead to a constant feeling of loss of control.

Most participants were aware of the high penetrance and hereditary aspect of LFS and were fully adherent in their participation in the surveillance program, matching results from previous studies in which individuals with inherited cancer risk had a positive attitude toward surveillance (Gopie et al., 2012). However, intense surveillance has proved to be burdensome for individuals with LFS (Jhaveri et al., 2015) adding to the high FoP scores in our participants. After initial diagnosis, FoP persists over time and is additionally triggered by situations such as follow‐up appointments (Koch et al., 2013; Simard et al., 2013). Our results indicate an association between the period that has passed since individuals learn of their LFS diagnosis (and have therefore experienced more examinations and appointments) and a more negative impact on their HRQoL. Previous research mostly focused on the time around genetic testing (Alderfer et al., 2017; Bakhuizen et al., 2019; Lammens, Aaronson, et al., 2010; Warby et al., 2019). However, the results of our study clearly indicate that there is a need for psychosocial support beyond the initial diagnosis to support adults in coping with LFS.

### 4.2 Distress

Nearly 70% of our sample presented clinically relevant levels of distress. This was unforeseen, as P/LP *TP53* VC reported lower levels of distress overall in a previous study on psychological burden. In that study, a high level of distress was associated with experiencing other distressful events in life (e.g. early losses of affected relatives, miscarriages) besides LFS (Peters et al., 2016). In our cohort, the emotional problems domain was most prominent, indicating that it contributes more to the overall distress of individuals with LFS than physical or practical problems (Table 4). Patients with only a suspected cancer diagnosis were at least as negatively affected as cancer patients after their diagnosis was confirmed (Brocken et al., 2012). This indicates that negative effects owing to a constant feeling of insecurity with regard to the possibility of cancer occurring can also be expected in our cohort.

### 4.3 Health‐related quality of life

Our P/LP *TP53* VC exhibited an impairment of HRQoL, especially mental, which is more comparable to that of long‐term cancer survivors (Chu et al., 2016) than that of cancer patients (Erim et al., 2020). The application of the cut‐off previously used in a sample of young cancer survivors therefore seems to be adequate (Harju et al., 2018). Although 67% of individuals in our sample had had cancer before, only 35% exhibited poor levels of physical health. Only 55% followed the recommended cancer surveillance program for early cancer detection. This might possibly indicate that adherence to surveillance leads to a reduction in physical impairment and that the motivation of affected individuals to adhere to surveillance programs is of great importance.

Our regression models demonstrated a poor fit. Neither demographic factors nor personal cancer history were significant, suggesting that psychosocial support is needed for the majority of people with LFS, despite their education, marital status, personal cancer history, or adherence to surveillance programs. LFS might be present in individuals' daily life constantly, in contrast to persons with non‐hereditary cancers. FoP was significantly associated with MCS in our sample, as in cancer patients with no underlying CPS (Sarkar et al., 2014).

On the one hand, CPS is associated with medical (e.g. medical appointments, personal cancer scares) and familial (e.g. traumatic familial cancer memories) uncertainties (Dean, 2016). On the other hand, surveillance might provide a source for a sense of control and security (Lammens, Bleiker, et al., 2010) and a way to remain in contact with professionals in order to stay informed on medical progress relating to LFS (Gopie et al., 2012; Ross et al., 2017). These different aspects of surveillance may be complex to balance, and be different for each individual. Female sex and younger age have often been identified as predictors of FoP (Custers et al., 2018; Koch et al., 2013; Simard et al., 2013). Especially for members of this demographic group, hereditary cancer (risk) is seen as a threat to pending life projects (Simard et al., 2013). There was a large percentage of young women in our sample, which may have contributed to the elevated levels of FoP recorded in our study. Furthermore, the limited variance of our study population may have eliminated the predictive effects of age and sex.

Cancer history did not significantly contribute to MCS in our sample. We thus hypothesize that knowing about and handling LFS itself is already burdensome, as patients with or without previous malignancies have valid reasons to worry about developing cancer. However, cancer history does contribute to PCS. Our results imply that HRQoL decreases with increasing time interval since the initial LFS diagnosis. On the one hand, the probability of developing cancer or recurrence increases with time, influencing PCS. On the other hand, any fear of developing cancer may also build up over time, impairing MCS. Providing psychosocial support on HRQoL to survivors and “previvors” with LFS is indicated, as it is a relevant factor of adherence to surveillance programs and cancer survival (Montazeri, 2009).

### 4.4 Strengths and limitations

Previous studies mainly focused on genetic testing or adherence to surveillance programs (Alderfer et al., 2017; Peterson et al., 2008; Warby et al., 2019). In contrast, we investigated a more general LFS population to provide cross‐sectional insight into the psychosocial burden of living with LFS (Eijzenga et al., 2014; Gopie et al., 2012). Despite the low prevalence, we still managed to recruit 70 individuals. Other studies focusing exclusively on LFS are scarce, as – owing to its rarity – research tends to observe LFS and other CPS simultaneously (Forbes Shepherd et al., 2018). We managed to stress the unique struggles P/LP *TP53* VC appear to face, because their options of prevention are comparably limited, given the multiple possible entities and highly elevated cancer risk (Peterson et al., 2008). So far, mainly explorative methods such as interviews (Alderfer et al., 2017; Ross et al., 2017; Valdez et al., 2017) or case studies (Bester et al., 2018; Jhaveri et al., 2015; Oppenheim et al., 2001) have been employed. We wished to address these through our quantitative approach and adapted the validated version of the FoP‐Q‐SF with good reliability (Eijzenga et al., 2014).

We also identified a number of limitations in our study. Owing to our recruitment strategy, we were unable to determine the reasons for not participating and have no information on the specific recruitment method with which individuals were selected. There are some variables we did not assess, which might nevertheless be moderators of psychosocial burden in *TP53* VC, namely the percentage of de novo participants, whether participants were currently undergoing cancer treatment, current psychosocial support, and critical life events (Peters et al., 2016). The poor fit of our regression models indicates that other factors influence HRQoL in adults with LFS. The variance in our sample was limited and the results possibly affected by selection bias. For example, the majority of participants adhered to the surveillance program. This may be due to the fact that a large proportion were recruited in the context of clinical appointments and were likely more accustomed to dealing with their syndrome actively. Furthermore, there are also factors limiting the generalizability of our study: We were unable to include individuals who disregarded or ignored the issue, as these do not frequently attend clinical appointments or participate actively in community groups. Such individuals may have different levels of perceived cancer risk or anxiety (Ritvo et al., 2002). Moreover, we assessed neither citizenship nor race in our study. Owing to limited variance, we were also not able to contribute to addressing the lack of information on the long‐term psychological impact of a diagnosis of LFS (Warby et al., 2019).

### 4.5 Practical implications

The highly prevalent FoP and distress in our cohort highlight a pressing need for psychosocial interventions. Our results suggest that there are no significant differences between survivors and “previvors.” Any specific psychosocial intervention can address the two groups simultaneously, thus increasing efficacy. FoP does not tend to decrease over time and can be a chronic issue (Simard et al., 2013). As research into LFS is lacking, we need to rely on existing psychosocial therapeutic concepts for cancer patients and survivors for the time being. Acceptance and commitment therapy (ACT) and cognitive behavioral therapy (CBT) are two evaluated methods to cope with fear, nervousness, symptoms of depression, distress, or grief. However, as our scores of FoP illustrate, *TP53* VC have particular needs resulting from their elevated risk and familial burden in comparison to non‐CPS cancer patients and survivors. We suggest using psychosocial CBT/ACT‐based interventions specifically and exclusively designed for LFS and comparable CPS participants to disclose their needs and fears. Providing specific educational content on genetics and inheritability, for example, may constitute a useful adaptation. Furthermore, including several family members and employing a group setting may prove beneficial.

A CBT approach evaluated on cancer patients previously reduced FoP by focusing on fears and the acquisition of coping strategies (Herschbach, Book, et al., 2010). A stepwise exposure to fears *in* sensu, psychoeducation, worry protocol, and identification of personal resources may be suitable (Dinkel, 2018; Rudolph et al., 2018).

We need to identify individuals with an elevated psychosocial burden resulting from LFS, as the proportion seems to be higher than that estimated in previous research. We thus recommend screening for psychosocial factors regularly, most efficiently as part of periodic surveillance appointments (Gopie et al., 2012; Lammens, Aaronson, et al., 2010). As part of a stepped care model, the previously described psychosocial interventions can be offered when screening indicates a need. This would also help to address the lack of research on the long‐term psychological impact of LFS diagnosis, as psychological burden probably fluctuates over time, with events in life and medical history often proving influential.

## 5 CONCLUSION

P/LP *TP53* VC exhibited high levels of FoP and distress, which are associated with a substantial impairment of the physical and particularly mental QoL in adults with LFS. Although some seem to cope well, we still stress the importance of screening for FoP and distress, for instance during routine medical check‐ups. There is a need to provide psychosocial support for adults with LFS beyond genetic testing or actual cancer diagnosis – a gap in health care that has not yet been addressed. How to provide psychosocial support to P/LP *TP53* VC who choose not to undergo genetic testing or refuse to adhere to cancer surveillance programs also remains an issue.

## AUTHOR CONTRIBUTIONS

SK: Writing: Original draft, data curation, formal analysis, investigation; SS: conceptualization, project administration, funding acquisition, resources, supervision, Writing: review and editing; JN: Writing: review and editing, investigation, data curation; CD: data curation, Writing: review and editing; FS: Writing: review and editing; CPK: conceptualization, project administration; CS: Writing: review and editing, formal analysis; AF: Writing: review and editing; MK: Writing: review and editing, investigation; IM: conceptualization, project administration, funding acquisition, resources, supervision, Writing: review and editing. Authors IM and SK confirm that they had full access to all the data in the study and take responsibility for the integrity of the data and the accuracy of the data analysis. All of the authors gave final approval of this version to be published and agree to be accountable for all aspects of the work in ensuring that questions related to the accuracy or integrity of any part of the work are appropriately investigated and resolved.

## FUNDING INFORMATION

SS and IM were supported by the Bundesministerium für Bildung und Forschung, Federal Ministry of Education and Research, BMBF (01GM1909D). CPK was supported by the Deutsche Kinderkrebsstiftung (DKS2019.13) and BMBF ADDRess (01GM1909A). JN was supported by the Faculty of Medicine in Heidelberg in the form of the Rahel‐Goitein‐Strauss Fellowship.

## CONFLICT OF INTEREST STATEMENT

SK, SS, JN, CD, FS, CPK, CS, AF, MK, and IM declare no conflict of interest.

## ETHICS STATEMENTS

Human Studies and Informed Consent: Approval to conduct this research on human subjects was obtained from the institutional review board of University Hospital Heidelberg and Hannover Medical School. All procedures followed were in accordance with the ethical standards of the responsible committee on human experimentation (institutional and national) and with the Helsinki Declaration (revised 2013). Informed consent was obtained from all patients prior to their inclusion in the study.

Animal studies: No non‐human animal studies were carried out by the authors for this article.

## Data Availability

The data that support the findings of this study are available from the corresponding author upon reasonable request.
